# Umbilical venous catheter- and peripherally inserted central catheter-associated complications in preterm infants with birth weight < 1250 g: Results from a survey in Austria and Germany

**DOI:** 10.1007/s10354-022-00952-z

**Published:** 2022-08-08

**Authors:** Steffi Hess, Martin Poryo, Ralf Böttger, Axel Franz, Daniel Klotz, Knud Linnemann, Torsten Ott, Johannes Pöschl, Michael Schroth, Anja Stein, Elisabeth Ralser, Heiko Reutter, Ulrich H. Thome, Christian Wieg, Anne Ehrlich, Christian Ruckes, Stefan Wagenpfeil, Michael Zemlin, Cihan Papan, Arne Simon, Johannes Bay, Sascha Meyer

**Affiliations:** 1 Department of General Pediatrics and Neonatology, Saarland University Medical Center, Kirrberger Str., Building 9, 66421 Homburg, Germany; 2 Department of Pediatric Cardiology, Saarland University Medical Center, Homburg, Germany; 3 University Children’s Hospital, Otto von Guericke University Magdeburg, Magdeburg, Germany; 4 University Children’s Hospital, Eberhard Karls University Tübingen, Tübingen, Germany; 5 Department of Neonatology, Center for Pediatrics, Medical Center, Faculty of Medicine, University of Freiburg, Freiburg, Germany; 6 University Children’s Hospital, Greifswald University Hospital, Greifswald, Germany; 7 University Children’s Hospital, University Hospital Muenster, Münster, Germany; 8 University Children’s Hospital, Heidelberg University Hospital, Heidelberg, Germany; 9 Children’s Hospital Nürnberg, Nürnberg, Germany; 10 University Children’s Hospital, Essen University Hospital, Essen, Germany; 11 University Children’s Hospital, Medical University of Innsbruck, Innsbruck, Austria; 12 Division of Neonatology and Pediatric Intensive Care Medicine, Department of Pediatric and Adolescent Medicine, Friedrich-Alexander-University Erlangen-Nürnberg, Erlangen, Germany; 13 University Children’s Hospital, University of Leipzig Medical Center, Leipzig, Germany; 14 Children’s Hospital Aschaffenburg, Aschaffenburg, Germany; 15 Interdisziplinäres Zentrum für Klinische Studien (IZKS), Johannes Gutenberg-Universität, Mainz, Germany; 16 Institute for Medical Biometry, Epidemiology and Medical Informatics, Saarland University Medical Center, Homburg/Saar, Germany; 17 Department of Medical Microbiology and Hospital Hygiene, Saarland University Medical Center, Homburg, Germany; 18 Department of Pediatric Hematology and Oncology, Infectious Diseases, Saarland University Medical Center, Homburg, Germany

**Keywords:** Survey, Very low birth weight infants, Umbilical venous catheter, Peripherally inserted central catheter, Infection, Thrombosis, Emboli, Organ injury, Umfrage, Säuglinge mit sehr niedrigem Geburtsgewicht, Nabelvenenkatheter, Peripher eingeführter Zentralkatheter, Infektion, Thrombose, Embolie, Organverletzung

## Abstract

**Background and objective:**

Umbilical venous catheters (UVC) and peripherally inserted central catheters (PICC) are commonly used in preterm infants but have been associated with a number of serious complications. We performed a survey in Austria and Germany to assess the use of UVCs and PICCs in preterm infants with a birth weight < 1250 g and associated rates of catheter-related adverse events.

**Methods:**

Electronic survey of participating centers of the NeoVitaA trial. Main outcome parameter was the reported rates of UVC- and PICC-associated complications (infection, thrombosis, emboli, organ injury, arrhythmia, dislocation, miscellaneous).

**Results:**

In total, 20 neonatal intensive care units (NICU) providing maximal intensive care in Austria and Germany (level I) were contacted, with a senior neonatologist response rate of 12/20 (60%). The reported rates for UVC with a dwell time of 1–10 days were bacterial infection: 4.2 ± 3.4% (range 0–10%); thrombosis: 7.3 ± 7.1% (0–20%); emboli: 0.9 ± 2.0% (0–5%); organ injury: 1.1 ± 1.9% (0–5%); cardiac arrhythmia: 2.2 ± 2.5% (0–5%); and dislocation: 5.4 ± 8.7% (0–30%); and for PICCs with a dwell time of 1–14 days bacterial infection: 15.0 ± 3.4% (range 2.5–30%); thrombosis; 4.3 ± 3.5% (0–10%); emboli: 0.8 ± 1.6% (0–5%); organ injury: 1.5 ± 2.3% (0–5%); cardiac arrhythmia: 1.5 ± 2.3% (0–5%), and dislocation: 8.5 ± 4.6% (0–30%).

**Conclusion:**

The catheter-related complication rates reported in this survey differed between UVCs and PICCs and were higher than those reported in the literature. To generate more reliable data on this clinically important issue, we plan to perform a large prospective multicenter randomized controlled trial investigating the non-inferiority of a prolonged UVC dwell time (up to 10 days) against the early change (up to 5 days) to a PICC.

**Supplementary Information:**

The online version of this article (10.1007/s10354-022-00952-z) contains supplementary material, which is available to authorized users.

## Abstract (de)

**Hintergrund und Ziel:**

Nabelvenenkatheter („umbilical venous catheters“ [UVC]) und periphere zentrale Venenkatheter (PICC) werden häufig bei Frühgeborenen eingesetzt, sind jedoch mit einer Reihe von schwerwiegenden Komplikationen verbunden. In Österreich und Deutschland wurde eine Umfrage durchgeführt, um die Verwendung von UVC und PICC bei Frühgeborenen mit einem Geburtsgewicht < 1250 g und die damit verbundenen Raten von katheterbedingten unerwünschten Ereignissen zu bewerten.

**Methoden:**

Elektronische Befragung der teilnehmenden Zentren der NeoVitaA-Studie. Hauptergebnisparameter waren die gemeldeten Raten von UVC- und PICC-assoziierten Komplikationen (Infektion, Thrombose, Embolie, Organverletzung, Arrhythmie, Dislokation, Sonstiges).

**Ergebnisse:**

Insgesamt wurden 20 neonatale Intensivstationen (NICU) mit maximaler Intensivpflege in Österreich und Deutschland (Level I) kontaktiert, wobei 12/20 (60 %) von leitenden Neonatologen beantwortet wurden. Die gemeldeten Raten für UVC mit einer Verweildauer von 1 bis 10 Tagen waren bakterielle Infektionen: 4,2 ± 3,4 % (Bereich: 0–10 %); Thrombose: 7,3 ± 7,1 % (0–20 %); Embolie: 0,9 ± 2,0 % (0–5 %); Organverletzung: 1,1 ± 1,9 % (0–5 %); Herzrhythmusstörungen: 2,2 ± 2,5 % (0–5 %); und Dislokation: 5,4 ± 8,7% (0–30 %); und bei PICC mit einer Verweildauer von 1 bis 14 Tagen bakterielle Infektionen: 15,0 ± 3,4 % (Bereich: 2,5–30 %); Thrombose: 4,3 ± 3,5 % (0–10 %); Embolie: 0,8 ± 1,6 % (0–5 %); Organverletzung: 1,5 ± 2,3 % (0–5 %); Herzrhythmusstörungen: 1,5 ± 2,3 % (0–5 %) und Verrenkungen: 8,5 ± 4,6 % (0–30 %).

**Schlussfolgerung:**

Die in dieser Umfrage berichteten katheterbedingten Komplikationsraten unterschieden sich zwischen UVC und PICC und waren höher als die in der Literatur berichteten. Um zuverlässigere Daten zu diesem klinisch wichtigen Thema zu erhalten, ist eine große prospektive, multizentrische, randomisierte, kontrollierte Studie geplant, in der die Nichtunterlegenheit einer verlängerten UVC-Verweildauer (bis zu 10 Tage) gegenüber dem frühen Wechsel (bis zu 5 Tage) zu einem PICC untersucht werden soll.

## Introduction

Umbilical venous catheters (UVCs) and peripherally inserted central catheters (PICC) are commonly used to establish a secure central vascular route for delivery of parenteral nutrition and drugs to preterm or sick newborn infants. UVCs are usually inserted within the first few hours postnatally [1]. Evidence suggests that use of UVCs and PICCs rather than peripheral venous cannulas facilitates consistent delivery of parenteral nutrients to preterm infants and reduces the number of painful venipunctures [2–4]. Furthermore, correctly positioned UVCs terminate within the inferior vena cava, which has been shown to reduce the risk of subcutaneous extravasation injury caused by hyperosmolar solutions and medications [5]. As with other types of central vascular catheters (CVCs), use of UVCs and PICCs is associated with complications, i.e., infections, thrombosis, organ injury [6, 7]. Moreover, bloodstream infection (BSI) is the most common serious adverse event, with the reported incidence ranging from 3 to more than 20%, depending on the diagnostic criteria applied, the demographics of the population studied, and the use of preventive bundles [8–10]. In particular, very preterm and very low birth weight (VLBW) infants with catheter-related BSI are at higher risk for mortality and for a range of important morbidities including bronchopulmonary dysplasia (BPD), necrotizing enterocolitis (NEC), retinopathy of prematurity (ROP), prolonged hospitalization, and higher rates of adverse neurodevelopmental outcomes [11–14].

Other potentially serious complications of UVC/PICC use include thrombosis formation [15], thromboembolism, cardiac arrhythmias triggered by a malposition of the UVC/PICC tip, and accidental migration of the UVC/PICC tip into peritoneal, pleural, or pericardial spaces. The latter may cause ascites, pleural effusion, or cardiac tamponade. Malposition of a UVC within the portal venous system may result in portal vein thrombosis, hepatic tissue necrosis, and long-term liver dysfunction [16, 17].

Controversy surrounds the duration of placement (so-called “dwell time”) and the pertinent risk of UVC- and PICC-associated BSI and other complications in preterm infants. Observational studies estimate that in UVCs the risk of BSI increases with dwell times longer than 7 to 14 days [10, 18]. It is not certain, however, to what extent UVC/PICC use is an independent risk factor for BSI, or whether observed associations exist because infants with lower birth weight and gestational age, receiving more intensive and invasive support, are more likely to have a UVC in place longer [19]. The US Center for Disease Control and Prevention (CDC) Hospital Infection Control Practices Advisory Committee currently recommends that UVCs should be removed as soon as possible when no longer needed but can be used for up to 14 days if managed aseptically [8, 20].

Replacement of intravenous access is often performed using PICCs—if ongoing central access is needed—or via peripheral venous cannulas [6]. It is unclear, however, whether or how this strategy of sequential central line use affects the rates of BSI and other complications [7].

Of importance, during their NICU stay, VLBW/very low gestational age neonates (VLGAN) are subjected to numerous painful invasive procedures, most often venipuncture for blood sampling, insertion of venous access, and endotracheal suctioning [2]. A longer dwell time of UVCs may have a significant impact on catheter-associated infections but may also reduce painful invasive procedures by early alternative vascular accesses. In the long run, the most significant clinical effects of early pain exposure may be on neurodevelopment, contributing to later attention, learning, and behavioral problems in these vulnerable children [21]. Thus, reducing the number of painful invasive procedures has the potential to positively impact not only on long-term pain perception but also on important social competencies as well.

To provide current data on the use of CVC and the rate of CVC-related complications (UVC/PICC) in very premature neonates with a birth weight <1250 g, we performed an electronic survey in level I NICUs in Austria and Germany.

## Methods

This survey is part of a research project performed at our hospital investigating the safety of different UVC dwell times in VLBW infants with a gestational age at birth of < 30 weeks and/or a birth weight < 1250 g. In addition, we are currently performing a pilot RCT comparing a UVC dwell time of 1–7 days with a dwell time of 8–14 days in VLBW infants (*Deutsches Register Klinische Studien* [DRKS]; German Clinical Trials Registry: DRKS00022262). The study was approved by our local ethics committee (*Ethikkommission des Saarlandes*, Saarbrücken, Germany; no.: 07/2020) and the *Bundesinstitut für Arzneimittelsicherheit und Medizinprodukte* (BfArm: 01042020). Subsequently, the concept of the pilot study will be extended to a multi-center RCT in premature infants (gestational age < 30 weeks and/or birth weight < 1250 g; “UVC—You Will See” study). For a more accurate calculation of sample size in an upcoming RCT, the “UVC Study,” we performed a survey with regard to UVC- and PICC-associated rates of complications (most importantly infection, thrombosis, organ injury).

Senior, leading neonatologists of all 20 study centers who participated in the NeoVitaA Trial were contacted and provided with our electronic survey, and were asked to participate in the planned multi-center UVC—You Will See study. The NeoVitaA trial is a large randomized controlled trial to assess the efficacy and safety of neonatal vitamin A supplementation and our hospital is the leading center of the RCT [22]. Data collection at the local sites was at the discretion of the participating centers. Both use of local data sources as well as data from the German Neonatal Network (GNN) was possible. In case of non-existent data with regard to specific items in our survey (e.g., catheter-related organ injury), senior neonatal expert opinion was considered adequate, as was best-possible information.

### Statistics

Data are presented as median, standard deviation of the mean, ranges, frequency distribution, and percentage. Retrospective data were provided. Some centers reported ranges of the occurrences. In those cases, the mean of the range was used for further calculation. Because of the low numbers of included centers and non-availability of individual patient data, no formal statistical analysis was performed.

### Questionnaire

Supplemental online file 1 details the electronic questionnaire. The most relevant catheter-related complications included bacterial infection, thrombosis, emboli, organ injury, cardiac arrhythmias, dislocation, and others. Furthermore, we assessed the following items: frequency of use in NICU, lumen (single-, double-lumen catheters), dwell time (days), use of standardized operating procedures (SOPs), use of anticoagulation, imaging method for assessment of catheter positioning, correct position at first attempt, and removal of UVC/PICC with regard to number of established enteral feeds.

Clinical sepsis was defined as a condition with at least two signs of systemic inflammatory response (temperature > 38 °C or < 36.5 °C, tachycardia > 200/min, new onset or increased frequency of bradycardias or apneas, hyperglycemia > 140 mg/dl, base excess < −10 mval/l, changed skin color, increased oxygen requirements), one laboratory sign (e.g., C‑reactive protein > 20 mg/L, leukocytosis with an immature/total neutrophil ratio > 0.2), and the neonatologist’s decision to treat with anti-infective drugs for at least 5 days but no proof of causative agent in blood culture [23]. Blood culture-confirmed sepsis was defined as clinical sepsis with detection of a pathogen in blood culture. If coagulase-negative *staphylococci* (CoNS) were isolated as the single pathogen in one peripheral venous blood culture, two clinical signs and one laboratory sign were required for classification of CoNS sepsis [24].

## Results

In total, 12 senior, leading neonatologists returned a complete questionnaire (12/20; 60%) and 11 declared their intent to participate in the upcoming RCT. According to the results of our survey, it was common practice to either use UVC and/or PICC in very premature small neonates (11/12 centers). In our survey, more than 75% of NICUs used SOPs with regard to the use of UVCs/PICCs. Peripherally inserted central catheters were used more often in comparison to UVCs in participating NICUs, and X‑ray was the leading method for assessment of catheter positioning (Table 1). Interestingly, dwell times differed between UVCs (no longer than 10 days) and PICCs (up to 14 days). It was common practice to use double-lumen UVCs as well as anticoagulation (Table 1).

**Table 1:** Overview of the most important findings from our survey with regard to use of umbilical venous catheter (UVCs) and peripherally inserted central catheters (PICCs) in premature neonates with a birth weight (BW) < 1250 g

	Umbilical venous catheter (UVC)	Peripherally inserted central catheter (PICC)
Frequency of use in NICU	Not at all: 1/12 (8.3%)	Not at all: 0/12 (0.0%)
	< 25%: 5/12 (41.7%)	< 25%: 5/12 (41.7%)
	25–49%: 2/12 (16.7%)	25–49%: 1/12 (8.3%)
	50–74%: 1/12 (8.3%)	50–74%: 4/12 (33.3%)
	≥ 75%: 3/12 (25.0%)	≥ 75%: 7/12 (58.3%)
Lumen	Single-lumen: 3/12 (25.0%)	Single-lumen: 12/12 (100%)
	double-lumen: 7/12 (58.3%)	
	both: 2/12 (16.7%)	
Dwell time in days (d)	1–5 d: 7/12 (58.3%) 1–10 d: 5/12 (41.7%)	1–5 d: 2/12 (16.7%) 1–10 d: 3/12 (25.0%) > 10 d: 5/12 (41.7%) Missing datasets: 2/12 (16.7%)
Use of standard operating procedure	Yes: 10/12 (83.3%)	Yes: 9/12 (75.0%)
	No: 2/12 (16.7%)	No: 3/12 (25.0%)
Use of anticoagulation	Yes: 9/12 (75.0%)	Yes: 6/12 (50.0%)
	No: 3/12 (25.0%)	No: 6/12 (50.0%)
Imaging method for assessment of positioning	X‑ray (only): 5/12 (41.7%)	X‑ray (only): 12/12 (100%)
	X‑ray/sonography: 5/12 (41.7%)	
	Sonography (only): 0/12 (0.0%)	
	Missing datasets: 2/12 (16.7%)	
Correct position at first attempt	58.2 ± 15.9% (range: 35–95%)	56.4 ± 3.5% (range: 20–95%)
Rate of bacterial infection	4.2 ± 3.4% (range: 0–10%)	15.0 ± 3.4% (range: 2.5–30%)
Rate of thrombosis	7.3 ± 7.1% (range: 0–20%)	4.3 ± 3.5% (range: 0–10%)
Rate of emboli	0.9 ± 2.0% (range: 0–5%)	0.8 ± 1.6% (range: 0–5%)
Rate of organ injury	1.1 ± 1.9% (range: 0–5%)	1.5 ± 2.3% (range: 0–5%)
Rate of cardiac arrhythmias	2.2 ± 2.5% (range: 0–5%)	1.5 ± 2.3% (range: 0–5%)
Rate of dislocation	5.4 ± 8.7% (range: 0–30%)	8.5 ± 4.6% (range: 0–30%)
Rate of complications (miscellaneous)	0.7 ± 1.7% (range: 0–5%)	1.4 ± 2.4% (range: 0–5%)
Removal UVC/PICC with regard to number of enteral feeds	≥ 100 ml/d/kg BW: 1/12 (8.3%)	≥ 100 ml/d/kg BW: 1/12 (8.3%)
	≥ 120 ml/d/kg BW: 6/12 (50.0%)	≥ 120 ml/d/kg BW: 6/12 (50.0%)
	≥ 140 ml/d/kg BW: 2/12 (16.7%)	≥ 140 ml/d/kg BW: 2/12 (16.7%)
	≥ 160 ml/d/kg BW: 1/12 (8.3%)	≥ 160 ml/d/kg BW: 1/12 (8.3%)

*NICU* neonatal intensive care unit

Further main results from our survey, most importantly with regard to UVC- and PICC-associated complication rates, are detailed in Table 1. Of note, with regard to each item, most importantly catheter-related complications, a great range between participating centers was noted.

## Discussion

The data and conclusions presented in this short communication are based on the results from a survey including 12 level I NICUs with a response rate of 60% from senior neonatologists. While the data provided by participating centers are thought to be precise, some inaccuracies with regard to reported catheter-related complications cannot be excluded with certainty, most importantly due to the retrospectively estimated and summarized reporting by participating experts, which intrinsically entails the risk of both under- and over-reporting. Moreover, some imprecision cannot be excluded due to the limited number of reporting centers.

Unexpectedly, the reported rates of UVC- and PICC-associated severe complications were higher than previously reported in the literature [25–28], with substantially higher rates of catheter-related complications when PICCs were in use compared to the use of UVCs. Of note, Pet et al. also reported a high rate of PICC-associated complications (in approximately one third of PICCs), but these included less severe complications (phlebitis, oedema, and perfusion changes) [28].

The data from our survey tentatively indicate that when central venous access is required in the early postnatal period in very premature neonates with a birth weight < 1250 g, it is prudent to initiate vascular access by inserting a UVC when feasible. Albeit somewhat speculative, the higher rate of catheter-related complications in PICCs, most notably infections, compared to UVCs may be explained by the timing as well as technical differences and difficulties when inserting a PICC in very premature infants (i.e., PICC line insertion is a painful procedure causing involuntary movements of the preterm infant), as well as by differences in dwell time. While the three most commonly reported catheter-related complications were infections, thromboses, and catheter dislocations, it is important to note that the use of CVC in premature infants may also be associated with rare, life-threatening complications (e.g., air emboli causing cardiac ischemia) [29].

Given the potential for benefit and harm associated with timing of removal of the UVC (and PICC) in preterm neonates, a prospective RCT of early planned removal vs. later planned removal of the UVC is warranted. Based on the results of our survey and preliminary data from our pilot study (DRKS: DRKS00022262), as well as data reported in the literature, we plan an RCT (UVC—You Will See study) to assess the optimal dwell time of UVCs in preterm neonates. Our trial will enroll 562 infants (based on an event rate of 30%) with a birth weight < 1250 g and/or a gestational age < 30 weeks with the need for prolonged CVCs for delivery of parenteral nutrition and/or drugs. The UVC—You Will See study will address primarily the effects of later removal (6–10 days dwell time) vs. early planned removal (1–5 days dwell time) on the risk of CVC-related BSI, thrombosis/emboli, and organ injury. Further important outcome parameters include, among others, the number of painful, invasive procedures, X‑rays, and the use of antibiotics.

Our multi-center RCT has the potential to demonstrate that a longer dwell time (6–10 days) is not associated with an overall increased rate of catheter-related infections, catheter-related thrombosis, and/or organ injury. Of note, a longer dwell time would reduce the need for insertion of another central venous catheter (PICC) or insertion of a peripheral venous catheter, thus decreasing the number of painful invasive procedures as well as the number of radiographs, use of antibiotics, and costs/medical expenditures. Thus, the UVC—You Will See trial has the potential to significantly alter the treatment of this highly susceptible cohort.

The results from our pilot study will provide important preliminary data, and minor adjustments depending on the rate of adverse events may be necessary prior to initiating our multi-center RCT.

In conclusion, our survey provides important insights into the rate of catheter-related adverse events in very premature infants treated in large level I NICUs in Austria and Germany, and in conjunction with the results from our pilot study (UVC—You Will See; DRKS-ID: DRKS00022262) and data from the published literature, serves as a basis for the most accurate sample size calculation for our multi-center RCT. The results from this study will provide the neonatal community with new relevant data on this important issue and may be helpful in preventing and reducing catheter-related adverse events, given the widespread use of CVCs, most importantly UVCs, in this highly susceptible cohort [30, 31].

Undoubtedly, some shortcomings of our survey are of relevance. First, a potential selection bias with only 20 NICUs contacted and a response rate of 12 may have been at play. These sites are all participating centers in the NeoVitaA study, and therefore do not represent the full spectrum of neonatal intensive care of more than 150 tertiary NICUs in Austria and Germany. Second, data collection by participating centers was done in a retrospective manner. Third, the process of data extraction from participating centers was not fully standardized, and it was at the discretion of the senior neonatologist to generate local data (from local data sources or in conjunction with data from the German Neonatal Network [GNN]). Moreover, whenever necessary, expert opinion and assessment was also permitted. Therefore, some inaccuracies with regard to reported catheter-associated complications cannot be excluded with certainty. However, given the fact that the reported rates in this survey were higher than in the published literature and were comparable to the preliminary data from our pilot RCT (UVC—You Will See; unpublished data), our data may possibly provide a better estimate and more precise picture on this important issue, although over-reporting cannot be excluded with certainty.

## Supplementary Information

Supplemental file 1: Electronic questionnaire/survey (in German)

## Abbreviations

BPD: Bronchopulmonary dysplasia
BSI: Blood stream infection
CDC: Center for Disease Control
CoNS: Coagulase-negative Staphylococci
CVC: Central vascular catheters
DRKS: Deutsches Register Klinische Studien (German Clinical Trials Registry)
ELBW: Extremely low birth weight
NEC: Necrotizing enterocolitis
PICC: Peripherally inserted central catheter
RCT: Randomized controlled trial
ROP: Retinopathy of prematurity
SOP: Standardized operating procedure
UVC: Umbilical venous catheter
VLBW: Very low birth weight
VLGAN: Very low gestational age neonates
