# Supplementary material for: Umbilical venous catheter- and peripherally inserted central catheter-associated complications in preterm infants with birth weight < 1250 g: Results from a survey in Austria and Germany
Source: Wien Med Wochenschr. 2022 Aug 8;173(7-8):161–7. doi: 10.1007/s10354-022-00952-z (PMC10147741; doi:10.1007/s10354-022-00952-z)
Supplement: Supplementary file 1 — Supplemental file 1: Electronic questionnaire/survey (in German) [file 10354_2022_952_MOESM1_ESM.docx]

|  |  |  |
| --- | --- | --- |
|  |  |  |
|  |  |  |

Sehr geehrte Kolleginnen und Kollegen,

liebe Prüferinnen und Prüfer der NeoVitaA-Studie,

Im Anschluss an die NeoVitaA-Studie planen wir eine multizentrische randomisierte Studie zur optimalen Liegedauer eines NVKs bei FG (1-5 Tage vs. 6-10 Tage; siehe hierzu Anlage: Synopsis des DFG-Antrages). Für eine bessere Fallzahlkalkulation benötigen wir Angaben zur Komplikationsrate bei Anlage NVK und PICC/ZVEK, denn die publizierten Daten hierzu variieren erheblich (3-20%).

Den ausgefüllten Survey könnten Sie mir sehr gerne als PDFile per Email); idealerweise bis spätestens 15.09.2021 zukommen lassen.

Vielen Dank für Ihre Unterstützung und wir würden uns sehr freuen, wenn es uns gelingen sollte, die angedachte Studie zu realisieren und Sie und Ihr Zentrum als Teilnehmer dafür zu gewinnen.

Mit besten Grüßen

**Survey zur Komplikationsrate von NVK/ZVEK/PICC in neonatologischen Zentren**

**in Deutschland und Österreich**

Anschrift/Name des Zentrums:

| Wie häufig verwenden Sie Nabelvenenkatheter (NVK/UVC) bei Neonaten mit einem Geburtsgewicht < 1250 Gramm? | |
| --- | --- |
| - gar nicht - <25% - 25-49% - 50-74% - >74% | |
| Kommentar: | |
| Verwenden Sie ein- oder zwei-lumige NVK? | |
| - Ein-lumiger NVK - Zwei-lumiger NVK | |
| Kommentar: | |
| Wie lange beträgt bei Ihnen in der Regel die NVK-Liegedauer in Tagen? | |
| - 1-5 Tage - 1-10 Tage - > 10 Tage - ….. Tage | |
| Kommentar: | |
| Verwenden Sie eine SOP zur Anlage und Pflege von NVKs? | |
| - Ja - Nein - …… | |
| Kommentar: | |
| Benutzen Sie z.B. Heparin zur Antikoagulation bei Verwendung eines NVKs? (z.B. 3 i.E. Heparin/kgKG/h) | |
| - Ja - Nein - …… | |
| Kommentar: | |
| Wie überprüfen Sie die korrekte Lage eines liegenden NVKs? | |
| - ausschließlich radiologisch (Röntgen) - ausschließlich sonografisch - Kombination aus Röntgen und Sonografie - ……… | |
| Kommentar: | |
| Wie hoch ist die Rate der korrekten Lage beim ersten Versuch? | |
| ……………..[%] | |
| Kommentar: | |
| Wie hoch ist bei Ihnen schätzungsweise die NVK-assoziierte Komplikationsrate für | |
| Infektionen | [%] |
| Thrombosen | [%] |
| Embolien | [%] |
| Organverletzungen | [%] |
| Arrhythmien | [%] |
| Dislokationen | [%] |
| Sonstige Komplikationen | [%] |
| Kommentar: | |
| Wie häufig verwenden Sie ZVEKs/PICCs („Peripherally Inserted Central Catheters“) bei Neonaten mit einem Geburtsgewicht < 1250 Gramm? | |
| - gar nicht - <25% - 25-49% - 50-74% - >74% | |
| Kommentar: | |
| Wie lange beträgt bei Ihnen in der Regel die PICC-Liegedauer in Tagen? | |
| - 1-5 Tage - 1-10 Tage - > 10 Tage - ….. Tage | |
| Kommentar: | |
| Verwenden Sie eine SOP zur Anlage und Pflege von PICC? | |
| - Ja - Nein - …… | |
| Kommentar: | |
| Benutzen Sie z.B. Heparin zur Antikoagulation bei Verwendung eines PICCs? (z.B. 3 i.E. Heparin/kgKG/h) | |
| - Ja - Nein - …… | |
| Kommentar: | |
| Wie überprüfen Sie die korrekte Lage eines liegenden PICCs? | |
| - ausschließlich radiologisch (Röntgen) - ausschließlich sonografisch - Kombination aus Röntgen und Sonografie - ……… | |
| Kommentar: | |
| Wie hoch ist die Rate der korrekten Lage beim ersten Versuch? | |
| ……………..[%] | |
| Kommentar: | |
| Wie hoch ist bei Ihnen schätzungsweise die PICC-assoziierte Komplikationsrate für | |
| Infektionen | [%] |
| Thrombosen | [%] |
| Embolien | [%] |
| Organverletzungen | [%] |
| Arrhythmien | [%] |
| Dislokationen | [%] |
| Sonstige Komplikationen | [%] |
| Kommentar: | |
| Wenn Sie sowohl NVK und PICC benutzen, wie hoch ist bei Ihnen schätzungsweise die damit verbundene kumulative Komplikationsrate? | |
| \| Infektionen \| [%] \| \| --- \| --- \| \| Thrombosen \| [%] \| \| Embolien \| [%] \| \| Organverletzungen \| [%] \| \| Arrhythmien \| [%] \| \| Dislokationen \| [%] \| \| Sonstige Komplikationen \| [%] \| \| Kommentar: \| \| | |
| Ab welcher enteraler Nahrungsmenge werden in Ihrem Zentrum zentrale Gefäßkatheter entfernt? | |
| \| Nahrungsmenge \| ≥ 100 ml/kgKG/d \| \| --- \| --- \| \| Nahrungsmenge \| ≥ 120 ml/kgKG/d \| \| Nahrungsmenge \| ≥ 140 ml/kgKG/d \| \| Nahrungsmenge \| ≥ 160 ml/kgKG/d \| \| Kommentar: \| \| | |
| Haben Sie grundsätzlich Interesse, an der von uns geplanten multizentrischen Studie „UVC – You Will SEE Study“ teilzunehmen? (DFG-Antrag anbei; zur Zeit in Überarbeitung befindlich) | |
| - Ja - Nein - …… | |
| Kommentar: | |
| Haben Sie Anregungen/Verbesserungsvorschläge für die angedachte „UVC – You Will See Study“? | |
| - Ja - Nein - …… | |
| Kommentar: | |

………………………………………………………………………………………………………………

Datum Name Unterschrift
